# 육아휴직 후 복직 간호사의 양육스트레스, 일-가정 갈등, 자아탄력성이 재직의도에 미치는 영향

**DOI:** 10.4069/kjwhn.2022.01.07

**Published:** 2022-03-18

**Authors:** Young-Eun Jung, Mi-Hae Sung

**Affiliations:** 1 Maternal-Fetal Intensive Care Unit, Busan Paik Hospital, Busan, Korea; 1 부산백병원 간호부, 고위험 산모·태아 집중치료실; 2 College of Nursing, Institute of Health Science, Inje University, Busan, Korea; 2 인제대학교 간호대학, 건강과학연구소

**Keywords:** Conflict, Parental leave, Resilience, Retention, Stress, 갈등, 육아휴직, 자아탄력성, 재직, 스트레스

## Abstract

**Purpose:**

This study investigated whether parenting stress, work-family conflict, resilience affect retention intent in Korean nurses returning to work after parental leave.

**Methods:**

The participants in this study were 111 nurses recruited from 10 hospitals in Korea, who were working after formal parental leave from their hospitals. Collected data were analyzed using descriptive statistics, the t-test, analysis of variance, the Mann-Whitney U-test, Pearson correlation coefficients, and hierarchical regression.

**Results:**

Retention intention (33.80±7.78), parenting stress (101.70±17.57), and resilience (85.02±12.75) were at greater than moderate levels and a midpoint level of work-family conflict (29.63±7.00) was noted in this sample of mostly women nurses in their 30s. The factors affecting retention intent were parental leave duration, the number of times that participants had taken parental leave, health condition, work-family conflict, and resilience. The total explanatory power of these variables was 36.7%. Retention intent had a negative correlation with parenting stress and work-family conflict. Conversely, retention intent was positively correlated with resilience

**Conclusion:**

This study supports the need for flexible adjustment of returning nurses’ working hours and family-friendly policies to promote balance between work and family. It is also necessary to develop and apply measures that boost resilience and support health improvement for nurses returning to work.As nurses are often assigned to new areas of work upon return, training programs to aid their adjustment may also be helpful.

## Introduction

결혼, 출산 및 육아로 인한 근로자의 이직을 막기 위한 일-가정 양립정책의 일환으로 우리나라 정부는 1987년부터 육아휴직 제도를 시행하고 있다[1]. 직군에 상관없이 전체 근로자 중 육아휴직을 사용하는 비율은 2009년 35,400명에서 2019년 105,165명으로 10년 사이에 약 3배 증가하였다[2]. 간호사의 경우 육아휴직은 184개(86.4%) 병원에서 제공하고 있으며 최소 2일에서 최대 1,825일까지 평균 457.2일을 제공하고 있다[3]. 이러한 배경 속에서 육아휴직 후 복직한 간호사들은 공백 기간 동안 변화된 의료 환경과 간호업무 재적응 및 자녀 양육의 문제로 어려움을 겪고 있다[4]. 육아휴직에서 복직한 간호사들은 질 높은 간호를 제공할 수 있는 경력 간호사들이 다수이므로[5], 복직 환경을 용이하게 하여 간호 조직에 긍정적 성과를 창출할 필요가 있다. 간호사의 이직률은 2019년 평균 15.4%이며 이직의 사유로 ‘결혼과 출산 및 육아’는 이중 8.5%를 차지하고 있다[6]. 간호사 보유율 증가를 위한 현재까지 연구 동향을 살펴보면 이직의도를 측정하는 것에 중점을 두어 왔다[7]. 그러나 이직과 같은 부정적인 결과와 연관된 이직의도와 달리 재직의도는 긍정적인 면을 가지고 있기 때문에[8], 재직의도를 파악하는 것이 지속해서 근무할 수 있는 환경 조성을 위한 실제적이고 효과적인 접근 방법이라고 할 수 있다[9]. 따라서 육아휴직 후 복직 간호사의 재직의도에 영향을 주는 요인을 파악하여 강화시킬 필요가 있다.

육아휴직 후 복직 간호사의 양육스트레스는 재직의도에 영향을 주지만[10,11], 양육스트레스가 재직의도에 직접적인 영향을 주지 않는다는 상반된 연구 결과[9]가 있어 이에 대한 더 많은 연구들이 지속되어야 할 것이다. 육아휴직 후 복직 간호사는 직장 중심적인 국내 근로 환경의 분위기에서[12] 가사와 자녀 양육의 문제를 담당해야 하므로 직장인, 엄마, 아내 등의 다양한 역할 요구 사이에서 일-가정 갈등을 경험한다[13]. 일-가정 갈등은 간호사의 재직의도에 영향을 미치는 요인으로 확인되어[14] 육아휴직 후 복직 간호사의 일-가정 갈등이 높을 것으로 예상되나, 이들의 일-가정 갈등과 재직의도와의 관계를 알아본 연구는 부족한 실정이다. 간호사를 대상으로 자아탄력성을 조사한 연구[15]에서 자아탄력성은 간호사의 재직의도에 긍정적인 요인으로 작용한다. 그러나 자아탄력성이 육아휴직 후 복직 간호사의 재직의도에 미치는 영향을 탐색한 연구는 국내‧외적으로 미비한 실정이기에 그 관계를 명확히 파악할 필요가 있다.

지금까지 육아휴직 후 복직 간호사를 대상으로 한 재직의도 관련 연구는 양육스트레스, 일-가정 갈등과 재직의도를 부분적으로 포함하여 진행한 연구뿐이다[9,11]. 따라서 본 연구에서는 육아휴직 후 복직 간호사들을 대상으로 양육스트레스, 일-가정 갈등, 자아탄력성과 재직의도의 정도 및 이들 변수들 간의 관계를 파악하고 재직의도에 미치는 영향요인을 규명함으로써, 이들의 업무 복귀 환경개선을 통하여 재직의도를 높이는 시도를 하여 효율적인 간호인력 관리와 더 나아가서 간호의 질적 향상에 필요한 기초자료를 제공하고자 한다.

## Methods

Ethics statement: This study was approved by the Institutional Review Board of Inje University (2020-10-003-001). Informed consent was obtained from the participants.

### 연구 설계

본 연구는 육아휴직 후 복직 간호사의 재직의도에 영향을 미치는 요인을 확인하기 위한 상관성 조사 연구로, STROBE (Strengthening the Reporting of Observational Studies in Epidemiology) 가이드라인을 따라 기술하였다[16].

### 연구 대상

본 연구의 대상은 부산광역시와 울산광역시에 소재한 총 10개의 병원에서 육아휴직을 실시한 후에 복직하여 근무하기 시작한지 1년 이하인 간호사를 대상으로 하였다. 복직 후 1년 이하인 자로 제한을 둔 것은, 육아휴직에서 복직 후의 업무 적응기간으로 94.4%가 ‘1년 이하’라고 응답한 연구 결과[5]를 근거로 하였다.

본 연구를 위해 필요한 표본 크기는 G power 3.1.9.4 program을 사용하였고 효과 크기 .15 (중간 크기), 유의 수준 .05, 검정력 .80이었다. 선행연구[11]에 근거하여 예측 변수는 8개(임상경력, 가정의 월평균 소득, 건강상태, 총 자녀 수, 육아휴직 후 복직한 이유, 양육스트레스, 일-가정 갈등, 자아탄력성)였다. 선행연구[11]에 근거하여 위계적 회귀분석에 필요한 최소 표본 수는 109명으로 산출했으나, 탈락률 15%를 고려하여 총 131명을 대상으로 하였다. 배부된 131부 중 120부(91.6%)를 회수하여 응답이 불충분한 9부를 제외한 111부를 최종 분석에 활용하였다.

### 연구 도구

본 연구에 사용한 모든 도구는 도구 개발자 및 번안자의 허락을 받았으며 필요 시 도구를 구입 후 사용하였다.

#### 재직의도

재직의도 측정은 Cowin [17]이 개발하고 Kim [18]이 한글로 표준화한 측정도구를 사용하였다. 총 6문항으로 구성된 8점 Likert 척도이며(전혀 그렇지 않다 1점, 매우 그렇다 8점), 점수 범위는 6–48점으로 점수가 높을수록 재직의도가 높음을 의미한다. 도구의 신뢰도 Cronbach’s α는 Kim [18]의 연구에서 .88, 본 연구에서는 .89였다.

#### 양육스트레스

양육스트레스 측정은 Kim과 Kang [19]이 취업모를 대상으로 개발한 양육스트레스 측정도구를 사용하였다. 총 32문항으로 자녀 양육으로 인한 일상적 스트레스 12문항, 부모 역할에 대한 부담감 및 디스트레스 12문항, 타인 양육에 대한 죄책감 8문항으로 구성되어 있는 5점 Likert 척도이며(전혀 그렇지 않다 1점, 매우 그렇다 5점), 점수 범위는 32–160점으로 점수가 높을수록 양육스트레스가 높음을 의미한다. 도구의 신뢰도 Cronbach's α는 Kim과 Kang [19]의 연구에서 .88, 본 연구에서는 .92였다.

#### 일-가정 갈등

일-가정 갈등 측정은 Netemeyer 등[20]이 직장인을 대상으로 개발한 일-가정 갈등 척도를 Kwon과 Choi [21]가 한글로 표준화한 측정도구를 사용하였다. 총 10문항으로 일-가정 방향 갈등 5문항, 가정-직장 방향 갈등 5문항으로 구성된 5점 Likert 척도이며(전혀 그렇지 않다 1점, 매우 그렇다 5점), 점수 범위는 10–50점으로 점수가 높을수록 직장 및 가정생활로 인한 갈등이 높음을 의미한다. 도구의 신뢰도 Cronbach’s α는 Kwon과 Choi [21]의 연구에서 .89, 본 연구에서는 .86이었다.

#### 자아탄력성

자아탄력성 측정은 Connor와 Davidson [22]이 개발한 Connor-Davidson Resilience Scale을 Baek 등[23]이 수정 보완한 측정도구를 사용하였다. 총 25문항으로 강인함 9문항, 인내력 8문항, 낙관성 4문항, 지지 2문항, 영성 2문항으로 구성된 5점 Likert 척도이며(전혀 그렇지 않다 1점, 매우 그렇다 5점), 점수 범위는 25–125점으로 점수가 높을수록 대상자의 자아탄력성 정도가 높음을 의미한다. 도구의 신뢰도 Cronbach’s α는 Baek 등[23]의 연구에서 .93, 본 연구에서는 .92였다.

### 자료 수집

자료 수집은 2021년 1월 11일부터 3월 10일까지 시행하였다. 본 연구자가 해당 병원 간호부의 승인을 받은 후 병원에 방문하여 대상자에게 연구의 취지와 목적을 설명하고 자발적인 동의를 구한 후 설문 조사를 실시하였다. 설문지의 작성 시간은 약 10–15분 정도 소요되었고 연구에 참여한 모든 대상자에게 소정의 선물을 제공하였다.

### 자료 분석 방법

수집된 자료는 IBM SPSS for Windows ver. 26.0 (IBM Corp., Armonk, NY, USA)을 이용하여 분석하였으며 분석 자료의 정규성 검정은 왜도와 첨도를 이용하였다. 구체적인 분석방법은 다음과 같다.

• 대상자의 일반적 특성과 육아휴직 관련 특성은 빈도와 백분율, 평균과 표준편차로 분석하였다.

• 대상자의 양육스트레스, 일-가정 갈등, 자아탄력성, 재직의도는 평균과 표준편차로 분석하였다.

• 대상자의 일반적 특성과 육아휴직 관련 특성에 따른 재직의도 차이는 independent t-test, 일원분산분석, Mann-Whitney U-test, 사후 검정은 Scheffé test로 분석하였다.

• 대상자의 양육스트레스, 일-가정 갈등, 자아탄력성, 재직의도의 상관관계는 Pearson correlation coefficients로 분석하였다.

• 대상자의 재직의도에 영향을 미치는 요인은 위계적 회귀분석으로 분석하였다.

## Results

### 대상자 특성에 따른 재직의도 차이

대상자의 일반적 특성 중 성별은 여성이 96.4% (107명), 연령은 평균 33.70±3.60세로 31–35세가 63.1% (70명)였다. 학력은 대학교 졸업이 55.0% (61명), 임상 경력은 평균 132.63±45.91개월로 120–179개월이 54.1% (60명), 근무 부서는 병동이 28.8% (32명), 현 부서 경력은 평균 56.81±53.60개월로 12개월 이하가 37.8% (42명)으로 많았다. 근무 직위는 일반 간호사가 73.9% (82명), 근무 형태는 3교대가 61.3% (68명), 가구의 월 수입은 평균 565.13±179.44만 원으로 400만 원 이하가 24.3% (27명), 건강상태는 ‘보통’이 46.8% (52명), 수면의 질은 ‘보통’이 42.3% (47명)로 많았다. 대상자의 육아휴직 관련 특성 중 육아휴직을 받은 횟수는 1회가 69.4% (77명), 육아휴직 이용기간은 평균 15.83±7.23개월로 12개월 이하가 58.6% (65명), 자녀 수는 1명이 61.3% (68명)로 많았으며, 근무 시 주 양육자는 보육시설이라고 응답한 대상자가 37.8% (42명), 퇴근 후 주 양육자는 본인이라고 응답한 대상자가 73.9% (82명)로 많았다. 복직 이유가 육아휴직 최대 기간 만료라고 응답한 대상자가 33.3% (37명)이었고 복직 시 근무지 이동이 없다는 응답이 51.4% (57명)로 많았다(Table 1).

대상자의 재직의도에 차이를 보이는 특성은 건강상태(F=16.40, *p*<.001), 육아휴직을 받은 횟수(t=4.01, *p*<.001), 육아휴직 이용기간(F=3.99, *p*=.021), 자녀 수(t=3.39, *p*=.001)였다(Table 1). 이를 Scheffé test로 사후 검정을 실시한 결과, 건강상태가 좋을수록 재직의도가 높게 나타났고 육아휴직 이용기간이 12개월 이하인 경우가 육아휴직 이용기간이 13–23개월인 경우보다 재직의도가 높았다. 그리고 육아휴직을 받은 총 횟수가 1회인 경우, 총 자녀 수가 1명인 경우가 육아휴직을 받은 총 횟수가 2회 이상인 경우, 총 자녀 수가 2명 이상인 경우보다 재직의도가 높았다.

대상자의 재직의도는 총 33.80±7.78점(6-48점 기준)으로 중등도 이상이었다. 양육스트레스는 총 101.70±17.57점(32-160점 기준)으로 중등도 이상, 일-가정 갈등은 총 29.63±7.00점(10-50점 기준) 으로 중등도였으며, 자아탄력성은 총 85.02±12.75점(25-125점 기준) 으로 중등도 이상이었다(Table 2).

### 대상자의 제 변수 간의 상관관계

대상자의 재직의도는 양육스트레스(r=–.29, *p*=.002), 일-가정 갈등(r=–.44, *p*<.001)과 약한 음의 상관관계가 있었고, 자아탄력성(r=.33, *p*<.001)과 약한 양의 상관관계가 있었다. 양육스트레스와 자아탄력성 간에는 약한 음의 상관관계(r=–.28, *p*=.002), 일-가정 갈등과 자아탄력성 간에는 약한 음의 상관관계(r=–.35, *p*<.001)가 있었으며, 양육스트레스와 일-가정 갈등 간에는 중강도로 양의 상관관계(r=.62, *p*<.001)가 있었다(Table 3).

### 대상자의 재직의도에 영향을 미치는 요인

대상자의 재직의도에 영향을 미치는 요인을 분석하기 위하여 위계적 회귀분석으로 일반적 특성 중 재직의도에 유의한 차이를 보인 건강상태와 육아휴직 관련 특성 중 육아휴직을 받은 횟수, 육아휴직 이용기간, 자녀 수와 재직의도에 유의한 상관관계를 보인 양육스트레스, 일-가정 갈등, 자아탄력성을 단계별로 투입한 결과는 Table 4와 같다. 본 연구의 위계적 회귀분석 결과 Durbin-Watson의 통계량은 1.522로 자기 상관의 문제가 없었고, 공차 한계(tolerance)는 .14–.87로 .10 이상의 값이 나타났으며 분산팽창지수(variance inflation factor)를 구한 결과 1.14–6.96로 10 미만의 값으로 나타났기에 회귀 모형은 적합하다고 할 수 있다.

제1회귀 모형(F=16.40, *p*<.001)의 설명력은 21.9%였으며, 통계적으로 유의한 변수는 건강상태 나쁨(β=–.32, *p*=.001), 건강상태 좋음(β=.26, *p*=.005) 순이었다. 제2회귀 모형(F=8.34, *p*<.001)의 설명력은 28.6%로 6.7% 증가하였으며, 건강상태 나쁨(β=–.30, *p*=.001), 건강상태 좋음(β=.25, *p*=.007), 육아휴직을 받은 횟수 1회(β=.48, *p*=.008), 육아휴직 이용기간 12개월 이내(β=–.51, *p*=.016), 육아휴직 이용기간 13개월 이상 24개월 미만(β=–.31 *p*=.083), 자녀 수(β=–.09 *p*=.538)로 나타나 통계적으로 유의한 변수는 육아휴직 이용기간 12개월 이내, 육아휴직을 받은 횟수 1회, 그리고 건강상태 순이었다. 제3회귀 모형(F=8.09, *p*<.001)의 설명력은 36.7%로 8.1% 증가하였으며, 건강상태 나쁨(β=–.29, *p*=.001), 건강상태 좋음(β=.15, *p*=.101), 육아휴직을 받은 횟수 1회(β=.37, *p*=.032), 육아휴직 이용기간 12개월 이내(β=–.45 *p*=.025), 육아휴직 이용기간 13개월 이상 24개월 미만(β=–.30 *p*=.075), 자녀 수(β=–.08 *p*=.547), 양육스트레스(β=.02, *p*=.785), 일-가정 갈등(β=–.24, *p*=.022), 자아탄력성(β=.18, *p*=.027)으로 통계적으로 유의한 변수는 육아휴직 이용기간 12개월 이내, 육아휴직을 받은 횟수 1회, 건강상태 나쁨, 일-가정 갈등, 자아탄력성 순으로 나타났다. 즉 육아휴직 이용기간이 짧을수록, 육아휴직을 받은 총 횟수가 적을수록, 건강상태가 좋을수록, 일-가정 갈등이 낮을수록, 그리고 자아탄력성이 높을수록 재직의도가 높아진다고 할 수 있다.

## Discussion

본 연구는 육아휴직 후 복직 간호사의 재직의도에 영향을 미치는 요인을 규명하고자 하였다. 본 연구 결과를 토대로 다음과 같이 논의하고자 한다.

본 연구에서 대상자의 재직의도에 영향을 미치는 요인은 육아휴직 이용기간, 육아휴직을 받은 횟수, 건강상태, 일-가정 갈등, 자아탄력성 순으로 나타났다. 이는 육아휴직 이용기간과 육아 휴직을 받은 횟수가 길어질수록 업무 공백이 생기고[11] 복귀 후 새로운 간호업무 수행의 어려움, 변화된 부서원들과의 친분과 팀워크 조성의 어려움 등을 겪기 때문으로 생각된다[4]. 이에 육아휴직 기간 동안 변경된 간호 업무와 신규 의료기기 사용법, 새로운 약물 교육 등과 관련한 교육 프로그램을 제공하여 복직 후 재적응 기간을 가질 수 있도록 하고, 직장 동료들과 새로운 팀워크를 잘 이끌어 나갈 수 있는 지지모임 등을 마련할 필요가 있다. 그리고 본 연구 결과에서 복직 시 근무지 이동은 재직의도에 영향을 미치지 않았지만, 복직 시 근무지 이동이 있다고 응답한 대상자가 46.8%이었고 육아휴직 후 직장에 복귀한 간호사의 적응 과정을 알아본 연구[4]에서 이들이 부서 배치에 대한 두려움을 경험하고 있는 것으로 나타났다. 이에 복직 시 근무지 이동 여부에 대하여 본인 의사를 반영하여 이들의 재적응을 도울 필요가 있다.

대상자의 재직의도에 대한 다른 영향요인은 건강상태로 나타났는데 이는 간호사의 재직의도에 미치는 영향을 분석한 연구[24]에서 건강상태가 재직의도에 영향을 준다는 결과와 일치한다. 이러한 결과로 볼 때 육아휴직 후 복직 간호사의 전반적인 정신적, 신체적 건강상태를 향상하는 것이 재직의도 제고에 효과적일 것으로 생각된다. 간호사 자신의 건강상태는 재직의도에 밀접한 관련이 있기에[24], 육아휴직 후 복직 간호사의 정신적, 신체적 건강상태를 주기적으로 평가하고 관리해주는 건강 증진 프로그램을 개발하여 제공하고 이들이 함께 고충을 나누고 감정을 표현할 수 있도록 동호회나 소모임에 참여할 수 있도록 지원하는 것이 필요하다.

대상자의 일-가정 갈등 또한 재직의도에 영향을 주는 요인으로 나타났는데 이는 육아휴직 후 복직 간호사의 재직의도 예측모형 구축에 관한 연구[9]에서 일-가정 갈등이 유의한 변수로 나타난 것과 일치하는 결과이다. 따라서 육아휴직 후 복직 간호사의 일-가정 갈등의 원인을 명확히 규명하는 것이 시급하며, 특히 교대 근무를 하는 경우 직장과 가정의 양립에 대한 갈등이 끊임없이 나타나므로[25] 이들이 양육시간에 맞추어 근무할 수 있도록 근로시간을 유연하게 사용할 수 있는 제도를 마련할 필요가 있고 동료 간호사들이 이를 배려하는 문화를 조성해야 한다.

본 연구에서 대상자의 재직의도에 대한 다른 영향요인은 자아탄력성으로 나타났는데, 이에 대한 선행연구가 없어 직접적인 비교는 어려운 실정이다. 자아탄력성이 높은 간호사는 전문적인 직업적 역량을 증진하고[26] 예술 치료는 자유롭게 감정을 표현하게 하여 자신감과 자존감을 향상할 수 있으므로[27], 육아휴직 후 복직 간호사를 대상으로 음악, 미술 치료 프로그램[28] 등과 같은 자아탄력성 증진 프로그램을 개발 및 제공하는 것이 필요하다.

한편 본 연구에서 대상자의 재직의도에 양육스트레스는 직접적인 영향을 주지 않았다. 이는 육아휴직 후 복직 간호사를 대상으로 한 연구[9]에서 양육스트레스가 재직의도에 직접적인 영향을 주지 않았다는 결과와 일치한 반면, 기혼 간호사를 대상으로 한 연구[10]에서 양육스트레스가 재직의도에 영향을 준다고 한 연구와 상반되는 결과이다. 하지만 본 연구에서 양육스트레스와 재직의도 사이에 유의한 음의 상관관계가 있었기 때문에 육아휴직 후 복직 간호사의 양육스트레스를 감소시켜 재직의도를 증가시킬 수 있는 방안이 필요하다고 판단된다.

본 연구에서 대상자의 재직의도는 양육스트레스, 직장-가정 갈등과 음의 상관관계가 있었고, 자아탄력성과 양의 상관관계가 있었다. 이는 육아휴직 후 복직 간호사를 대상으로 한 연구[9]에서 재직의도는 양육스트레스, 직장-가정 갈등과 음의 상관관계가 있다고 보고한 것이나, 간호사를 대상으로 한 연구[15]에서 재직의도는 자아탄력성과 양의 상관관계가 있는 것으로 나타난 결과와 일치한다. 따라서 대상자들의 재직의도를 높이기 위해서 자녀 양육에 있어 적극적인 가족의 참여와 보육시설 제공 및 자기 계발을 할 수 있는 기회가 요구된다.

이상의 논의를 통해 육아휴직 후 복직 간호사의 재직의도를 증진하기 위해서는 근로시간을 탄력적으로 조정하여 근무할 수 있도록 하고 가정 친화적인 정책을 구축 및 적용하여 직장과 가정을 양립할 수 있도록 하여야 한다. 그리고 자기 계발을 할 수 있는 기회 제공 및 자아탄력성 증진 프로그램 개발과 적용이 필요하다. 또한 육아휴직 동안 변경된 간호 업무 등과 관련된 교육 프로그램을 제공하여 복직 후 재적응 기간을 가질 수 있도록 하여야 한다. 그리고 이들의 정신적, 신체적 건강상태를 주기적으로 평가하고 관리해주는 건강증진 프로그램의 개발과 지원이 필요하다. 이를 통해 수준 높은 간호를 환자들에게 제공할 수 있고 육아휴직 후 복직 간호사의 재직의도를 높일 수 있을 것으로 생각되며 이를 탐색하는 후속 연구를 제안한다.

본 연구의 제한점은 연구대상이 일부 지역의 10개 병원을 편의 표집 조사 하였으므로 확대 해석에 신중을 기해야 하며 연구 대상자의 수가 최소 표본 수를 충족하였으나 총 분석에 활용한 대상자가 많지 않다는 점을 고려한 해석이 필요할 것으로 생각된다. 그러나 육아휴직 후 복직 간호사의 재직의도에 미치는 영향 요인을 분석하여 이들의 재직의도를 높일 수 있는 프로그램 개발과 정책 마련 및 체계를 구축해야 할 필요성을 제시하였다는 점에서 의의가 있다.

## Figures and Tables

**Table 1.** Differences in retention intent according to participants’ characteristics (N=111)

Variable	Categories	n (%)	Mean±SD	t or F	*p*
Sex	Female	107 (96.4)	33.69±7.90		.342^†^
	Male	4 (3.6)	36.75±1.70		
Age (year)		Mean±SD	33.70±3.60		
	≤30	16 (14.4)	36.25±8.88	0.88	.450
	31–35	70 (63.1)	33.28±7.88		
	36–39	15 (13.5)	34.80±6.82		
	≥40	10 (9.0)	32.00±7.78		
Educational level	College	39 (35.1)	34.66±7.49	0.63	.533
	University	61 (55.0)	34.04±8.04		
	≥Graduate school	11 (9.9)	34.90±7.62		
Clinical experience (month)		Mean±SD	132.63±45.91		
	≤60	7 (6.3)	31.14±8.74	1.05	.374
	61–119	24 (21.6)	35.83±7.24		
	120–179	60 (54.1)	33.78±7.88		
	≥180	20 (18.0)	32.35±7.75		
Work unit	General ward	32 (28.8)	34.37±6.17	1.42	.222
	Outpatient department	30 (27.0)	34.43±8.52		
	Intensive care unit	19 (17.1)	34.63±7.98		
	Emergency room	6 (5.4)	32.83±6.88		
	Operating room and anesthesiology	19 (17.1)	30.05±9.20		
	Delivery room	5 (4.5)	38.60±3.50		
Current clinical experience (month)		Mean±SD	56.81±53.60		
	≤12	42 (37.8)	33.40±6.73	0.16	.851
	13–95	32 (28.8)	34.43±8.24		
	≥96	37 (33.3)	33.70±8.64		
Position	Staff nurse	82 (73.9)	33.91±7.49	0.15	.875
	≥Charge nurse	29 (26.1)	33.64±8.80		
Work shift schedule	Only daytime	40 (36.0)	33.82±8.71	0.08	.922
	2-shift	3 (2.7)	32.00±4.35		
	3-shift	68 (61.3)	33.86±7.39		
Monthly family income (KRW)		Mean±SD	565.13±179.44		
	≤4 million	27 (24.3)	33.59±9.01	0.63	.638
	4.1 million-5 million	17 (15.3)	31.94±7.39		
	5.1 million-6 million	26 (23.4)	34.53±7.92		
	6.1 million-7 million	23 (20.7)	35.43±5.71		
	≥7 million	18 (16.2)	32.72±8.53		
Subjective health status	Poor^a^	23 (20.7)	27.47±8.25	16.4	<.001
	Moderate^b^	52 (46.8)	33.69±6.57		(a<b<c)^‡^
	Good^c^	36 (32.4)	38.00±6.34		
Sleep quality	Poor	46 (41.4)	33.67±7.49	0.15	.861
	Moderate	47 (42.3)	33.57±8.58		
	Good	18 (16.2)	34.72±6.57		
Number of times of parental leave	1	77 (69.4)	35.64±6.82	4.01	<.001
	≥2	34 (30.6)	29.54±8.42		
Duration of parental leave (month)		Mean±SD	15.83±7.23		
	≤12^a^	65 (58.6)	35.38±7.48	3.99	.021
	13–23^b^	40 (36.0)	31.10±8.14		(a>b)^‡^
	≥24^c^	6 (5.4)	34.66±2.65		
Number of children	1	68 (61.3)	35.70±6.87	3.39	.001
	≥2	43 (38.7)	30.79±8.26		
Main caregiver during work	Childcare facilities	42 (37.8)	33.85±5.84	1.17	.325
	Parent and relatives	34 (30.6)	32.58±8.97		
	Parents-in-law	18 (16.2)	32.83±1.06		
	Spouse	13 (11.7)	36.38±6.92		
	Babysitter	4 (3.6)	32.50±3.31		
Main caregiver after work	Self	82 (73.9)	33.43±7.69	1.21	.309
	Spouse	15 (13.5)	34.66±5.62		
	Parent and relatives	9 (8.1)	35.77±10.70		
	Parents-in-law	3 (2.7)	28.00±10.14		
	Childcare facilities	2 (1.8)	42.00±4.24		
Main reason for returning to work	Expiration of the maximum period	37 (33.3)	32.18±6.88	1.59	.169
	Economic difficulty	29 (26.1)	32.13±7.50		
	Opportunities for social activities	18 (16.2)	35.83±6.64		
	Self-actualization and development	14 (12.6)	35.71±11.61		
	Others	8 (7.2)	36.37±6.58		
	Getting out of housework	5 (4.5)	38.60±5.27		
Rotation to new workplace upon return	Yes	54 (46.8)	34.59±7.49	1.04	.300
	No	57 (51.4)	33.05±8.04		

† Mann-Whitney *U*-test,

‡ Scheffé test.

KRW: Korean won (1 million is approximately 900 US dollars).

**Table 2.** Participants' retention intent, parenting stress, work-family conflict, and resilience (N=111)

Variable	Possible score range	Mean±SD	Min	Max
Retention intent	6-48	33.80±7.78	6	48
Parenting stress	32-160	101.70±17.57	48	145
Work-family conflict	10-50	29.63±7.00	10	45
Resilience	25-125	85.02±12.75	52	120

**Table 3.** Correlations among retention intent, parenting stress, work-family conflict, and resilience (N=111)

Variable	r (*p*)		
	Retention intent	Parenting stress	Work-family conflict
Retention intent	1		
Parenting stress	–.29 (.002)	1	
Work-family conflict	–.44 (<.001)	.62 (<.001)	1
Resilience	.33 (<.001)	–.28 (.002)	–.35 (<.001)

**Table 4.** Factors influencing retention intent (N=111)

Variable	Model 1			Model 2			Model 3		
	B	β	t (*p*)	B	β	t (*p*)	B	β	t (*p*)
(Constant)	5.61		35.29 (<.001)	5.91		12.83 (<.001)	5.62		4.81 (<.001)
Subjective health status^†^									
Poor	–1.03	–.32	–3.60 (<.001)	–0.97	–.30	–3.37 (.001)	–0.95	–.29	–3.48 (.001)
Good	0.71	.26	2.88 (.005)	0.7	.25	2.76 (.007)	0.41	.15	1.65 (.101)
Number of times of parental leave^†^				1.36	.48	2.71 (.008)	1.04	.37	2.17 (.032)
Duration of parental leave (month)^†^									
≤12				–1.36	–.51	–2.45 (.016)	–1.19	–.45	–2.27 (.025)
13–23				–0.85	–.31	–1.75 (.083)	–0.82	–.30	–1.79 (.075)
Number of children^†^				–0.25	–.09	–.61 (.538)	–0.23	–.08	–.60 (.547)
Parenting stress							0.06	.02	.27 (.785)
Work-family conflict							–0.44	–.24	–2.3 (.022)
Resilience							0.47	.18	2.2 (.027)
F(*p*)	16.40 (<.001)			8.34 (<.001)			8.09 (<.001)		
Adjusted R²	.219			.286			.367		

† Reference variables were subjective health status (moderate), number of times of parental leave (≥2), duration of parental leave reference (≥24 months), and number of children (≥2).
